# ATXN10 Is Required for Embryonic Heart Development and Maintenance of Epithelial Cell Phenotypes in the Adult Kidney and Pancreas

**DOI:** 10.3389/fcell.2021.705182

**Published:** 2021-12-14

**Authors:** Melissa R. Bentley-Ford, Reagan S. Andersen, Mandy J. Croyle, Courtney J. Haycraft, Kelsey R. Clearman, Jeremy B. Foote, Jeremy F. Reiter, Bradley K. Yoder

**Affiliations:** ^1^ Department of Cell, Developmental and Integrative Biology, University of Alabama at Birmingham, Birmingham, AL, United States; ^2^ Department of Microbiology, University of Alabama at Birmingham, Birmingham, AL, United States; ^3^ Department of Biochemistry and Biophysics, University of Alabama at Birmingham, San Francisco, CA, United States; ^4^ Chan Zuckerberg Biohub, San Francisco, San Francisco, CA, United States

**Keywords:** ataxin 10 (ATXN10), epithelial-to-mesenchymal transition (EMT), cilia, acinar-to-ductal metaplasia (ADM), heart, kidney, pancreas

## Abstract

*Atxn10* is a gene known for its role in cytokinesis and is associated with spinocerebellar ataxia (SCA10), a slowly progressing cerebellar syndrome caused by an intragenic pentanucleotide repeat expansion. *Atxn10* is also implicated in the ciliopathy syndromes nephronophthisis (NPHP) and Joubert syndrome (JBTS), which are caused by the disruption of cilia function leading to nephron loss, impaired renal function, and cerebellar hypoplasia. How *Atxn10* disruption contributes to these disorders remains unknown. Here, we generated *Atxn10* congenital and conditional mutant mouse models. Our data indicate that while ATXN10 protein can be detected around the base of the cilium as well as in the cytosol, its loss does not cause overt changes in cilia formation or morphology. Congenital loss of *Atxn10* results in embryonic lethality around E10.5 associated with pericardial effusion and loss of trabeculation. Similarly, tissue-specific loss of ATXN10 in the developing endothelium (Tie2-Cre) and myocardium (cTnT-Cre) also results in embryonic lethality with severe cardiac malformations occurring in the latter. Using an inducible Cagg-CreER to disrupt ATXN10 systemically at postnatal stages, we show that ATXN10 is also required for survival in adult mice. Loss of ATXN10 results in severe pancreatic and renal abnormalities leading to lethality within a few weeks post ATXN10 deletion in adult mice. Evaluation of these phenotypes further identified rapid epithelial-to-mesenchymal transition (EMT) in these tissues. In the pancreas, the phenotype includes signs of both acinar to ductal metaplasia and EMT with aberrant cilia formation and severe defects in glucose homeostasis related to pancreatic insufficiency or defects in feeding or nutrient intake. Collectively, this study identifies ATXN10 as an essential protein for survival.

## Introduction

*Ataxin10* (*Atxn10*) is most commonly associated with spinocerebellar ataxia type 10 (SCA10), which is caused by an ATTCT pentanucleotide expansion within intron 9 (Matsuura et al., 2000). The consequences of the pentanucleotide expansion on ATXN10 function or nonexpansion coding mutations on the function of ATXN10 remain unclear. Investigation of the pentanucleotide expansion mutation indicates that the allele is transcribed at normal levels and is spliced normally (Wakamiya et al., 2006). To date, the only reported human incidence of *Atxn10* mutation (IVS8-3T > G) in the protein-coding region was observed in three Turkish siblings from a consanguineous family. This mutation resulted in Nephronophthisis-like kidney defects that ultimately led to death as infants (Sang et al., 2011). This same study further identified ATXN10 as a Nephronophthisis (NPHP)- and Joubert syndrome (JBTS)-associated gene that indirectly interacts with the ciliary transition zone protein, NPHP5, near the base of the cilium. NPHP is a form of medullary cystic kidney disease with associated nephron loss (Luo and Tao, 2018), while JBTS is autosomal recessive or X-linked cerebellar ataxia associated with cerebellar hypoplasia (Romani et al., 2013). Both NPHP and JBTS fall into the class of disorders collectively termed *ciliopathies*. Ciliopathies result from improper structure or function of the primary cilium. These small microtubule-based appendages are present on the surface of nearly every mammalian cell type and are crucial for mediating many cell signaling events (Sharma et al., 2008).

Knockdown of *Atxn10* in rat primary cortical and especially cerebellar neurons is cytotoxic (Marz et al., 2004). Interestingly, overexpression of *Atxn10* alone is sufficient to induce neuritogenesis in neuronal precursor cells where it interacts with the G-protein β2 subunit to drive activation of the RAS–MAPK–ELK-1 signaling cascade (Waragai et al., 2006). Furthermore, Aurora B phosphorylation of ATXN10 promotes its interaction with Polo Like Kinase 1 (Plk1) (Tian et al., 2015). This interaction between ATXN10 and Plk1 is necessary for cytokinesis *in vitro* (Li et al., 2011). The function of ATXN10 *in vivo* is largely unresolved.

To initiate studies into the *in vivo* functions of ATXN10, we established congenital (*Atxn10* ^*KO*^) and conditional (*Atxn10* ^*flox*^) mutant mice and assessed the consequence of ATXN10 loss during both embryogenesis and in adult tissues*.* Congenital loss of ATXN10 results in severe cardiac development abnormalities and gestational lethality. Tissue-specific ablation of ATXN10 in the developing endothelium and myocardium similarly results in embryonic lethality. Induction of ATXN10 loss in adult mice causes lethality likely due to moderate to severe pancreatic, renal, and gastrointestinal abnormalities, and severe defects in glucose homeostasis. Further analysis of renal phenotypes revealed an epithelial-to-mesenchymal transition (EMT) of the kidney tubule epithelial cells. Similarly, in the pancreas, acinar cells appear to undergo a transdifferentiation process resulting in more progenitor-like phenotypes.

Previous work indicates that ATXN10 is predominantly a cytoplasmic protein with cell cycle-dependent localization of the phosphorylated protein (on Serine 12) to the Golgi during interphase, the centrioles during prophase, and the midbody during telophase (Tian et al., 2015; Tian et al., 2017). Our studies similarly indicate that localization of ATXN10 is predominantly cytoplasmic; however, it also localizes near the centrioles and at the base of the primary cilium. While a ciliary role for ATXN10 cannot be excluded, we show that loss of ATXN10 does not affect ciliogenesis in fibroblast or epithelial cells, although acini in *Atxn10* postnatal-induced mutants do exhibit ectopic cilia possibly associated with their altered cell state.

## Results

### Loss of Ataxin 10 does not Affect Cilia Formation or Maintenance

**FIGURE 1:**
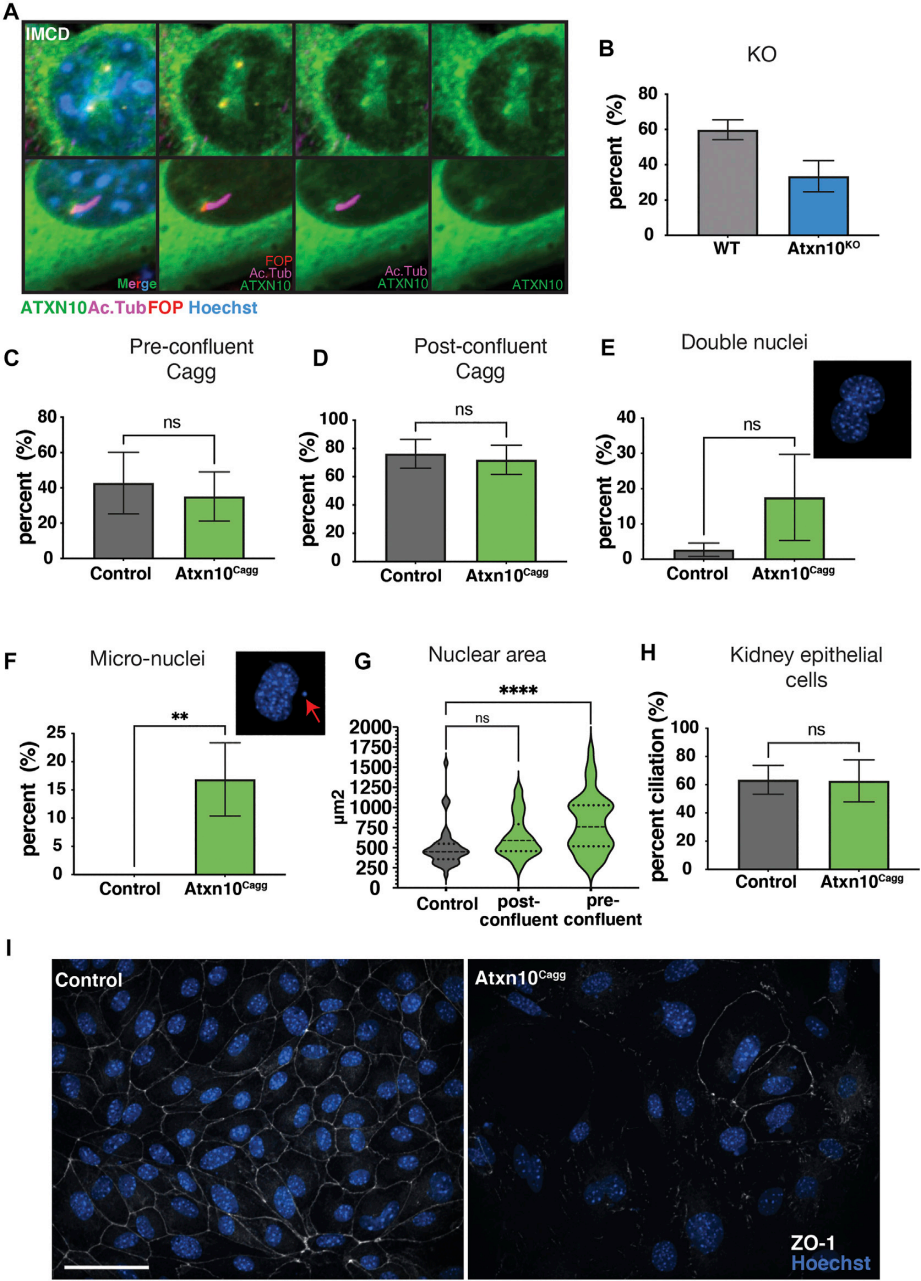
*In vitro* analysis. **(A)** Ataxin 10(ATXN10)-EGFP localized to the cilia basal body and centrioles in inner medullary collecting duct (IMCD) cells stained for cilia, Acetylated α-tubulin (Ac. Tub, purple) and basal bodies, FGFR1 oncogene partner (FOP, red), and hoechst (blue) for nuclei. **(B)** Percent ciliation in control (*N* = 2) and *Atxn10* ^*KO*^ mouse embryonic fibroblasts (MEFs) (*N* = 2). **(C)** Percent cilia in control (*N* = 6) and *Atxn10* ^*Cagg*^ MEFs (*N* = 6) when Cre is induced prior to confluency. **(D)** Percent cilia in control (*N* = 5) and *Atxn10* ^*Cagg*^ MEFs (*N* = 6) when Cre is induced after confluency. **(E, F)** Percent of control (*N* = 3) and *Atxn10* ^*Cagg*^ (*N* = 4) primary kidney epithelial cells containing two nuclei and exhibiting nuclear blebbing and micronuclei formation. **(G)** Nuclear area (µm^2^) in primary kidney epithelial cells in control, and induced post- and preconfluent cells. **(H)** Percent cilia in primary kidney epithelial cells from control (*N* = 3) and *Atxn10* ^*Cagg*^ (*N* = 4) animals that were postconfluent. **(I)** Immunofluorescence staining for tight junction protein, zonula occludens-1 (ZO-1, white) in noninduced (control) and induced mutant (*Atxn10* ^*Cagg*^) primary kidney epithelial cells (scale bar = 50 μm). Statistical significance of nuclear area was determined using a one-way ANOVA with multiple comparisons. All other statistical significance was determined using unpaired *t*-test. The number of biological replicates is indicated by N with at least 50 cells quantified for each replicate.

To determine the localization of ATXN10, we generated an EGFP-tagged ATXN10 (ATXN10-EGFP) for expression in cultured cells (attempts to detect endogenous ATXN10 with several commercial antisera were not successful). Overexpression of ATXN10-EGFP in inner medullary collecting duct (IMCD) cells supports a predominantly cytoplasmic expression pattern; however, enrichment of ATXN10-EGFP near FGFR1OP (FOP)-positive centrioles or basal bodies is seen in 79.3% of the transfected cells, regardless of whether they had a primary cilium. Of the transfected cells that have cilia, we detected an enrichment of ATXN10 around the base of the cilia (Figure 1A). This information led to the investigation of whether ATXN10 is necessary for ciliogenesis.

In mouse embryonic fibroblasts (MEFs) generated from *Atxn10* ^*KO*^ embryos, there was a trend toward fewer cilia, but these differences were not statistically significant between *Atxn10* ^*KO*^ (33.5%) and control (59.8%) cells (*p* = 0.07) (Figure 1B). MEFs generated from *Atxn10* ^*KO*^ embryos were unable to be maintained in culture for longer than two or three passages (see below). As the formation of the primary cilium is tied to the cell cycle (Malicki and Johnson, 2017), we wanted to determine whether the loss of ATXN10 affected ciliary maintenance following cilia formation. To address this question, we generated conditional MEFs using *Atxn10* ^*flox/flox*^; *Cagg-Cre* (*Atxn10* ^*Cagg*^) embryos induced ATXN10 loss prior to, or after, confluency and then serum starved to induce cilia formation. Regardless of whether induction occurred prior to confluency or postconfluency, removing ATXN10 in *Atxn10* ^*Cagg*^ MEFs did not compromise cilia (Figures 1C, D).

To observe the effect of ATXN10 loss in an epithelial cell line, primary kidney epithelial cells were isolated from *Atxn10* ^*Cagg*^ mice. Similar to what was observed in MEFs, loss of ATXN10 prior to confluency resulted in cells that failed to grow to confluency and could not be maintained. Comparatively, control cells grew to become confluent (data not shown). *Atxn10* ^*Cagg*^ primary renal epithelial cells trended toward having an increased prevalence of cells with two nuclei [17.5% in *Atxn10* ^*Cagg*^ compared with 2.7% in controls, (*p* = 0.09)] (Figure 1E). They also exhibited a large increase in nuclear blebbing and micronuclei formation (16.8% in *Atxn10* ^*Cagg*^ compared with 0% in controls) (Figure 1F), and nuclear size was increased in cells induced prior to confluency (*p* < 0.0001; with nuclear area in control cells averaging 504 μm^2^, preconfluent-induced cells averaging 632 μm^2^, and postconfluent-induced cells averaging 799 μm^2^) (Figure 1G). The frequency with which postconfluent-induced primary kidney epithelial cells presented a cilium was not different between control and *Atxn10* ^*Cagg*^ cells (63.4% in control cells versus 62.7% in *Atxn10* ^*Cagg*^ cells) (Figure 1H). Another consistent observation regarding the primary kidney epithelial cells was an increase in cell spreading and more fibroblast-like cell morphology following Cre induction of postconfluent cultures (Figure 1I and Supplementary Figure S1F). While these cells were plated at the same density following induction, fewer cells were frequently observed in mutant cell cultures than in controls. Thus, part of this phenotype may be related to cell density. Staining for the epithelial tight junction marker ZO-1 in postconfluent-induced cells shows a distinct loss of localization to the cell–cell junction in *Atxn10* ^*Cagg*^ primary kidney epithelial cells compared with noninduced controls (Figure 1I). Collectively, these findings support the role of ATXN10 in cell division. Although ATXN10 transiently accumulates around the ciliary basal bodies, it is dispensable for ciliogenesis. These data also indicate a potential role for ATXN10 in maintaining epithelial cell phenotypes like tight junctions.

### *Atxn10 in Vivo* Expression Analysis

**FIGURE 2:**
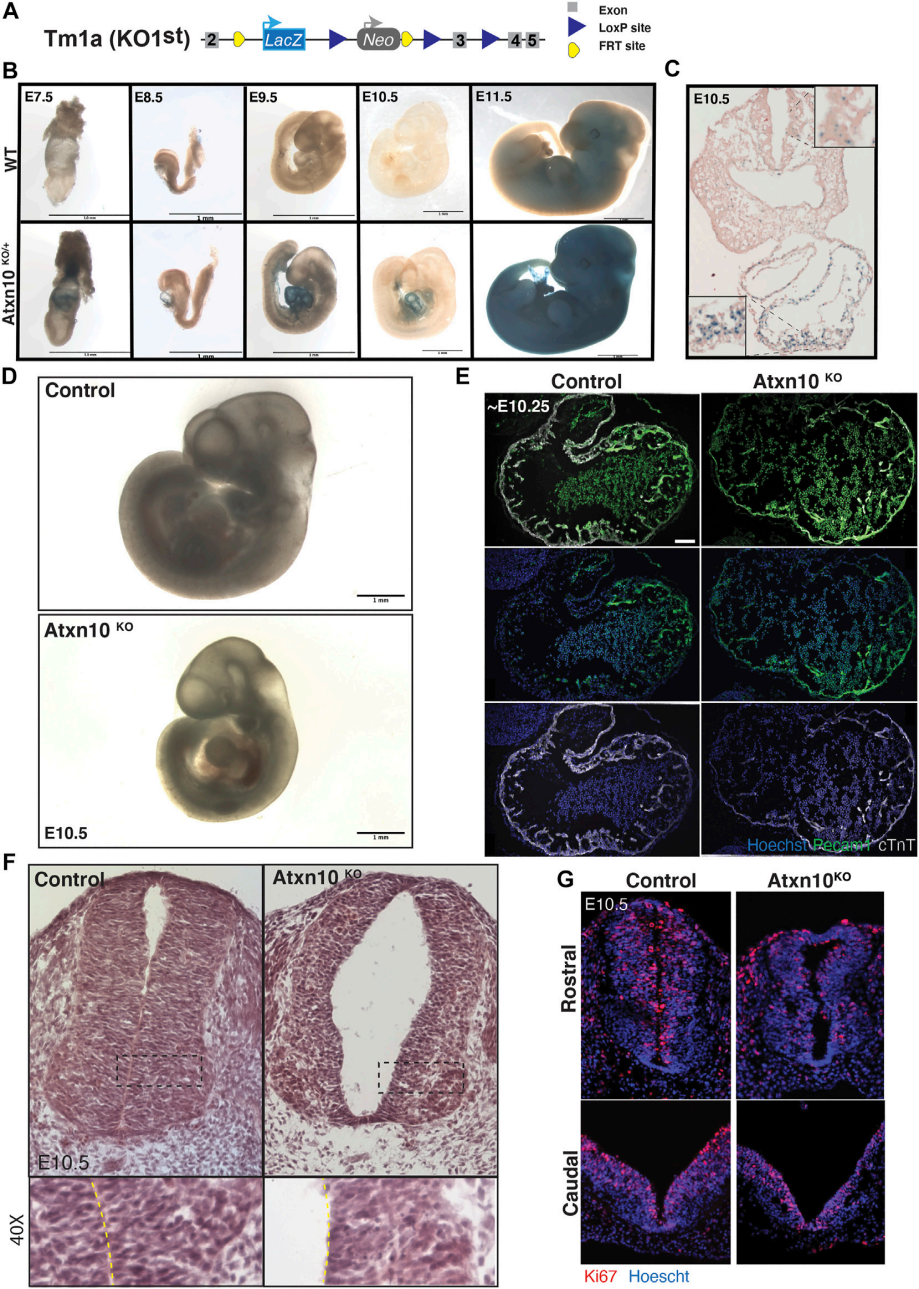
Phenotypic analysis of congenital loss of ATXN10. **(A)** Schematic depicting the *Atxn10* KO1st allele. **(B)** β-Galactosidase staining in wild-type (top) and heterozygous (bottom) animals at E7.5, E8.5, E9.5, E10.5, and E11.5. **(C)** β-Galactosidase staining of a transverse cross section of an E10.5 heterozygous embryo. Note the expression in the neuroepithelium of the neurotube in the inset. **(D)** Images of wild-type **(top)** and *Atxn10* ^*KO*^ **(bottom)** embryos at E10.5 (scale bar = 1 mm). **(E)** Immunofluorescence staining for PECAM1 (green), cTnT (white), and Hoechst (blue) in the heart of wild-type and *Atxn10* ^*KO*^ embryos at E10.5 (scale bar = 100 μm). **(F)** H&E staining of transverse sections from control and *Atxn10* ^*KO*^ rostral neural tubes at E10.5. **(G)** Ki67 (red) and nuclei (blue) staining of caudal **(top)** and rostral **(bottom)** neural tubes in WT and *Atxn10* ^KO^mutant animals.

The *Atxn10* promoter-driven expression of *LacZ* in the Tm1a knockout first (KO) allele (Figure 2A) allowed gene expression to be examined via β-galactosidase staining. Staining performed on the heterozygous *Atxn10* ^*KO/+*^ and wild-type control embryos indicates that *Atxn10* is highly expressed in the developing heart tube from embryonic day 8.5 (E8.5) to E10.5 with lower levels of positive staining in most tissues and regions of the embryo (Figures 2B,C). By E11.5, β-galactosidase staining becomes more prominent throughout the entire embryo. β-galactosidase staining performed on sections of E10.5 *Atxn10* ^*KO/+*^ embryos shows expression of *Atxn10* in both the myocardium and endocardium with positively stained cells present throughout the mesoderm and within the neural tube (Figure 2C). Several attempts were made to confirm the β-galactosidase staining pattern with that of endogenous ATXN10 expression as determined by *in situ* hybridization; however, none of the probes generated resulted in specific staining for ATXN10. Thus, the expression data must take this into account, but it should be noted that the β-galactosidase staining is consistent with the MGI expression analysis of *Atxn10* (MGI:1859293).

### Congenital Loss of Ataxin 10 Results in Pericardial Effusion and Embryonic Lethality

In agreement with the embryonic lethality reported by the International Mouse Phenotyping Consortium (IMPC), we found that *Atxn10* mutant embryos die after E10.5 (Wakamiya et al., 2006; Dickinson et al., 2016). Between E10.0 and E10.5, 6 out of 18 mutant embryos displayed gross morphological cardiac abnormalities or developmental delay compared with none out of the 20 wildtype and 49 heterozygous controls (Figure 2D). Immunofluorescence staining of sections through the heart region using markers for the endothelium (PECAM1) and the myocardium (cTnT) shows that while both layers are present in *Atxn10* ^*KO*^ embryos, the walls of the developing heart are thinned with an apparent reduction in trabeculation (Figure 2E).

### Congenital Loss of Ataxin 10 Results in Neural tube Defects

In addition to the cardiac defects mentioned above, structural abnormalities were also observed in the neural tubes of mutant embryos (Figures 2F,G). H&E staining of E10.5 embryos highlight the thin, disorganized structure and altered cell morphology of the neural epithelium (Figure 2F). Although the rostral neural tubes have closed, they exhibit an increase in size of the luminal space. While the caudal neural tube has not fully closed in the control nor mutant embryos, Ki67 staining in both rostral and caudal neural tubes indicate that the thinned appearance of the neural tube may result from a lack of proliferation in the mutant embryos (Figure 2G).

### Tissue-Specific Ablation of Ataxin 10 in the Myocardium and Endothelium Results in Embryonic Lethality

**FIGURE 3:**
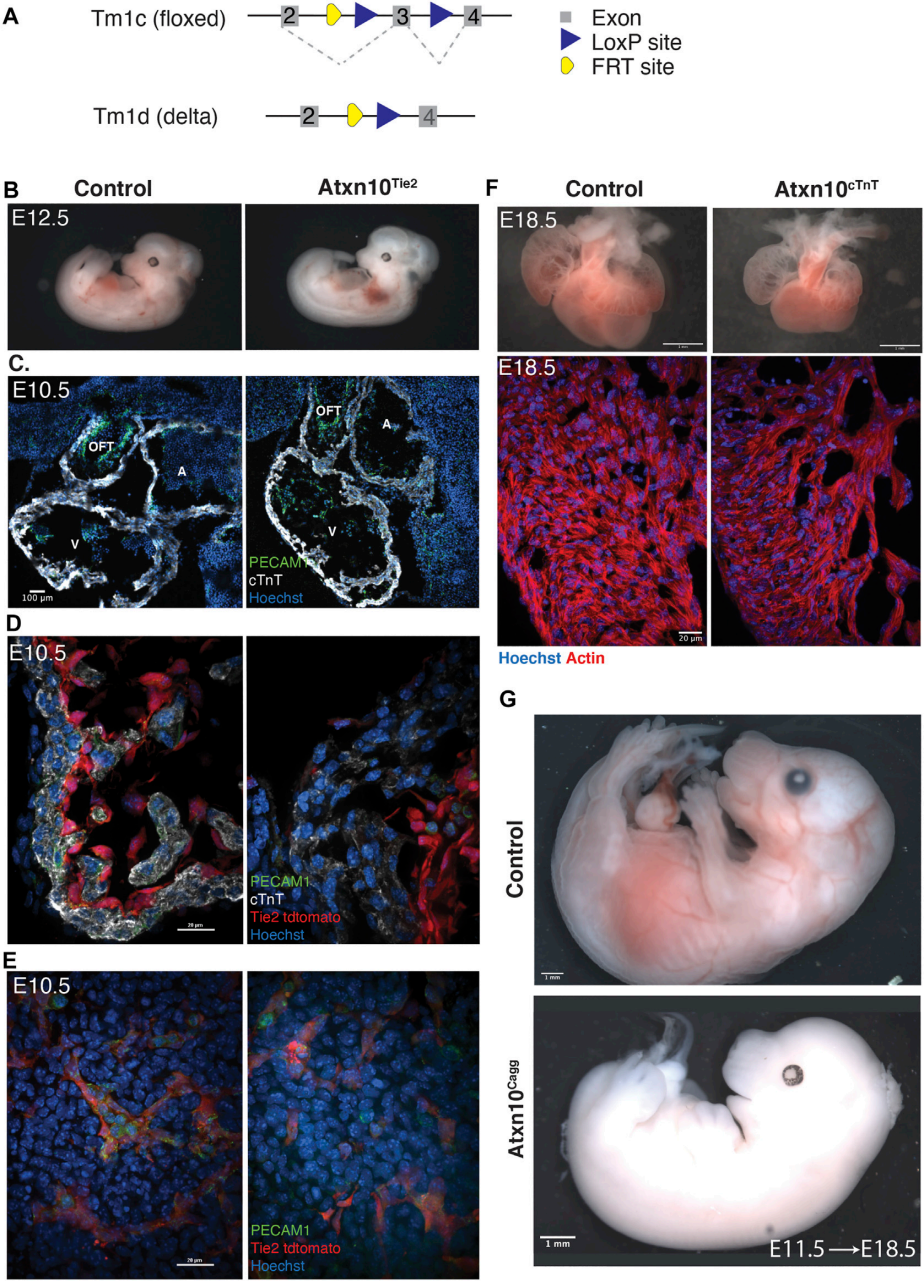
Tissue-specific ablation of ATXN10. **(A)** Schematic depicting the *Atxn10* tm1c (floxed) allele and schematic depicting the *Atxn10* tm1d (delta) allele. **(B)** Control and *Atxn10* ^*Tie2*^ mutant embryos at E12.5. **(C)** Immunofluorescence staining of sagittal cardiac cross sections taken from E10.5 control and *Atxn10* ^*Tie2*^ embryos with markers for PECAM1 (green), cTnT (white), and Hoechst (blue) (scale bar = 100 μm). **(D)** Immunofluorescence staining of cardiac trabeculae in the ventricles of E10.5 control and *Atxn10* ^*Tie2*^ embryos representing PECAM1 (green), representing cTnT (white), and nuclei (blue). Activity of Tie2-Cre is marked in red by tdTomato reporter inside the cardiac chambers (scale bar = 100 μm). **(E)** Immunofluorescence staining of vasculature in E10.5 mesoderm in control and *Atxn10* ^*Tie2*^ embryos with PECAM1 (green), cTnT (white), Hoechst (blue), and Tie2-Cre activity (red) are marked by tdTomato reporter (scale bar = 100 μm). **(F)** Hearts isolated from control and *Atxn10* ^*cTnT*^ embryos at E18.5 (scale bar = 1 mm). Immunofluorescence staining of ventricular trabeculae with actin (red) and nuclei (blue) (scale bar = 20 μm). **(G)** Images of control and *Atxn10* ^*Cagg*^ embryos induced *in utero* at E11.5 and isolated at E18.5 (scale = 1 mm).

At the stage that pericardial effusion is observed in *Atxn10* ^*KO*^ embryos, interactions between the developing endocardium and myocardium are crucial for proper development (Samsa et al., 2013). To determine if the cardiac phenotype seen in congenital knockout mice is specifically due to the loss of ATXN10 in the developing myocardium or endocardium, the conditional *Atxn10* allele was used (Figure 3A). By inducing loss of ATXN10 in the developing endothelium using the Tek2-Cre (Tie2) (Kisanuki et al., 2001; Koni et al., 2001), embryos exhibit lethality between E11.5 and E13.5. *Atxn10* ^*Tie2*^ embryos collected at E10.5 are grossly indistinguishable from control littermates (Supplementary Figure S2A). Embryos isolated at E12.5 can be distinguished from littermates due their pale coloration and pooling of blood around the heart (Figure 3B). Embryos that can still be recovered at E13.5 show a similar pale coloration indicative of cardiovascular or hemopoetic abnormalities (Supplementary Figure S2B). Staining of *Atxn10* ^*Tie2*^ mutants prior to death indicate that the endothelium is still present in both the developing heart (Figures 3C,D) and in the embryonic mesoderm (Figure 3E) indicated by the presence of PECAM1 and Tie2-Cre-positive vasculature.

Using a cTnT-Cre, we also assessed the effect of ATXN10 loss in the myocardium (Jiao et al., 2003). These mutants died perinatally; however, at E18.5, *Atxn10* ^*cTnT*^ embryos displayed edema and blood pooling (Supplementary Figure S2C). Closer observation of the heart indicates reduced trabeculation, ventricular noncompaction, and overall smaller size compared with controls (*N* = 10 control and *N* = 6 *Atxn10* ^*cTnT*^ embryos) (Figure 3F).

The spatiotemporal specificity of gene expression and the failure of the two tissue-specific mutants to phenocopy the congenital heart phenotype while still resulting in lethality led to the question of whether embryonic lethality is specific to cardiac abnormalities. To test this, timed matings were established between *Atxn10* ^*flox/flox*^ females and *Atxn10* ^*flox/flox*^ *Cagg-CreERT2*-positive males. Pregnant females were then induced 11.5 days into pregnancy. Seven (7) days following induction (E18.5), the resulting Cre-positive embryos were also nonviable (Figure 3G). Although these embryos had been induced following the initial cardiac morphological events that were impaired in *Atxn10* ^*KO*^ embryos, lethality was still ultimately the outcome supporting the conclusion that ATXN10 plays vital roles in processes beyond early cardiovascular development.

### Loss of Ataxin 10 in Adult mice Results in Pancreatic, Renal, and Gastrointestinal Abnormalities Followed by Abrupt Lethality

**FIGURE 4:**
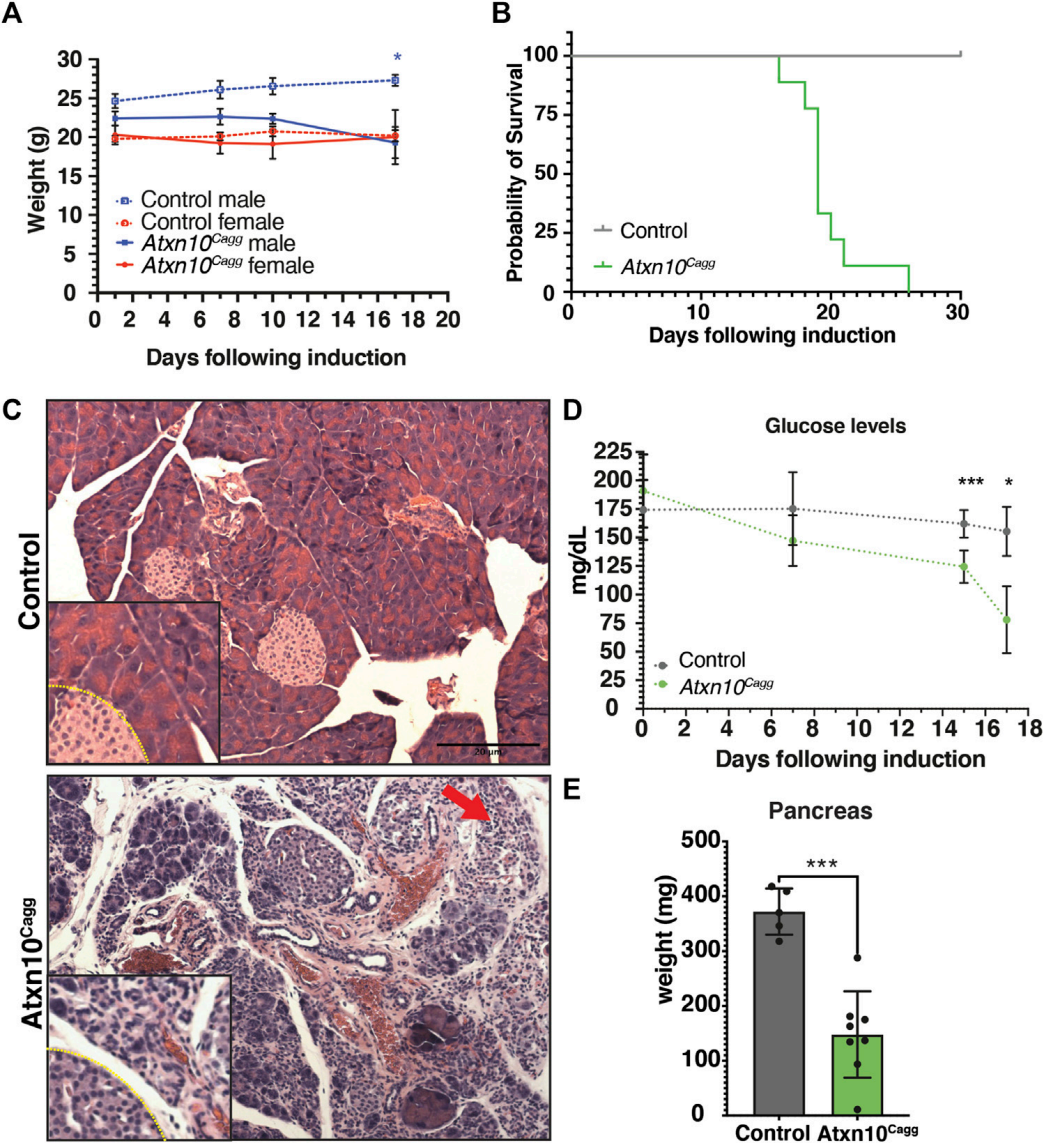
Pancreas defects associated with ATXN10 loss. **(A)** Weights following induction of control and *Atxn10* ^*Cagg*^ animals at 8 weeks of age (*p *= 0.047 in male mice at 17 days postinduction; *N* = 7 Cre− females, 2 Cre− males, 6 Cre+ females, and 4 Cre+ males). **(B)** Kaplan–Meier survival curve of control (gray) and *Atxn10* ^*Cagg*^ (green) animals induced at 8 weeks old (*p* < 0.0001; *N* = 9 Cre− and 9 Cre+ animals). **(C)** H&E staining of *Atxn10* ^*flox/flox*^ **(top)** and *Atxn10* ^*Cagg*^ **(bottom)** pancreas with a red arrow indicating acinar cell necrosis. **(D)** Altered levels of blood glucose in nonfasted wildtype and mutant mice. *p *= 0.002 at 15 days postinduction and *p* = 0.0399 at 17 days postinduction (*N* = 5 Cre− and 8 Cre+ up to 15 days postinduction and *N* = 3 Cre− and 4 Cre+ mice at day 17). No differences were evident at 6 days postinduction. **(E)** Pancreatic weight at 15–17 days postinduction (*p* = 0.0001, *N* = 5 Cre− and 8 Cre+ animals). Statistical significance was determined using mixed effects analysis with multiple comparisons for change in weight and change in glucose levels over time. Statistical significance of survival was determined via log-rank test. Statistical significance of pancreas weight was determined using unpaired *t*-test.

To determine whether ATXN10 is necessary postnatally, loss of ATXN10 was induced in *Atxn10* ^*Cagg*^ adult mice at 4 and 8 weeks of age with recombination of the conditional allele determined via analysis of genomic DNA and reduction of expression by rt-PCR (Supplementary Figure S3A). As expected, based on previous studies using the Cagg–CreER line and a single tamoxifen injection, complete deletion of the floxed alelle was not obtained. Even though we did not get complete deletion, this had dramatic effects. Male and female mice induced at 4 weeks of age failed to gain weight (Supplementary Figure S3B). When the animals were induced at 8 weeks of age, only male mice exhibited a significant weight difference at 17 days postinduction (Figure 4A). Strikingly, *Atxn10* ^*Cagg*^ (Cre positive) mice induced at 4 weeks (data not shown) and 8 weeks of age resulted in abrupt lethality between 16 and 26 days postinduction (Figure 4B). Pathological analysis of tissues isolated from control and *Atxn10* ^*Cagg*^ animals prior to death indicates that a major contributor to their death is most likely pancreatic acinar injury. This is supported by the histology of *Atxn10* ^*Cagg*^ pancreata showing cytoplasmic basophilia (an increase in cytoplasmic hematoxylin staining) and acinar cell necrosis with significant lymphocytic infiltrates within and around affected acini indicating a recent injury to the pancreas (Figure 4C). Analysis of serum glucose levels in nonfasted animals showed a significant decrease in blood glucose levels in *Atxn10* ^*Cagg*^ animals compared with control (Figure 4D). The pancreata in *Atxn10* ^*Cagg*^animals were significantly smaller in size relative to their controls (Figure 4E). Lethality is likely due to the combined effect of pancreatic damage and reduced food intake indicated by hyperkeratosis of the nonglandular region of the gastrointestinal tract (data not shown) and hepatocyte atrophy (Supplementary Figure S3D). Further analysis of the stomach in *Atxn10* ^*Cagg*^ animals revealed moderate lymphocytic and neutrophilic infiltrate of the submucosal and mucosal regions (data not shown). Additionally, in the stomach, mild crypt dilation, epithelial necrosis, and diffuse, mild epithelial hyperplasia, dysplasia, and mild mineralization indicated chronic, active gastritis, and secondary epithelial changes (data not shown). Interestingly, these indicators of an ongoing insult to the glandular epithelium were not observed in the nonglandular regions of the gastrointestinal tract (duodenum/jejunum, cecum/colon, and omentum/mesentery).

**FIGURE 5:**
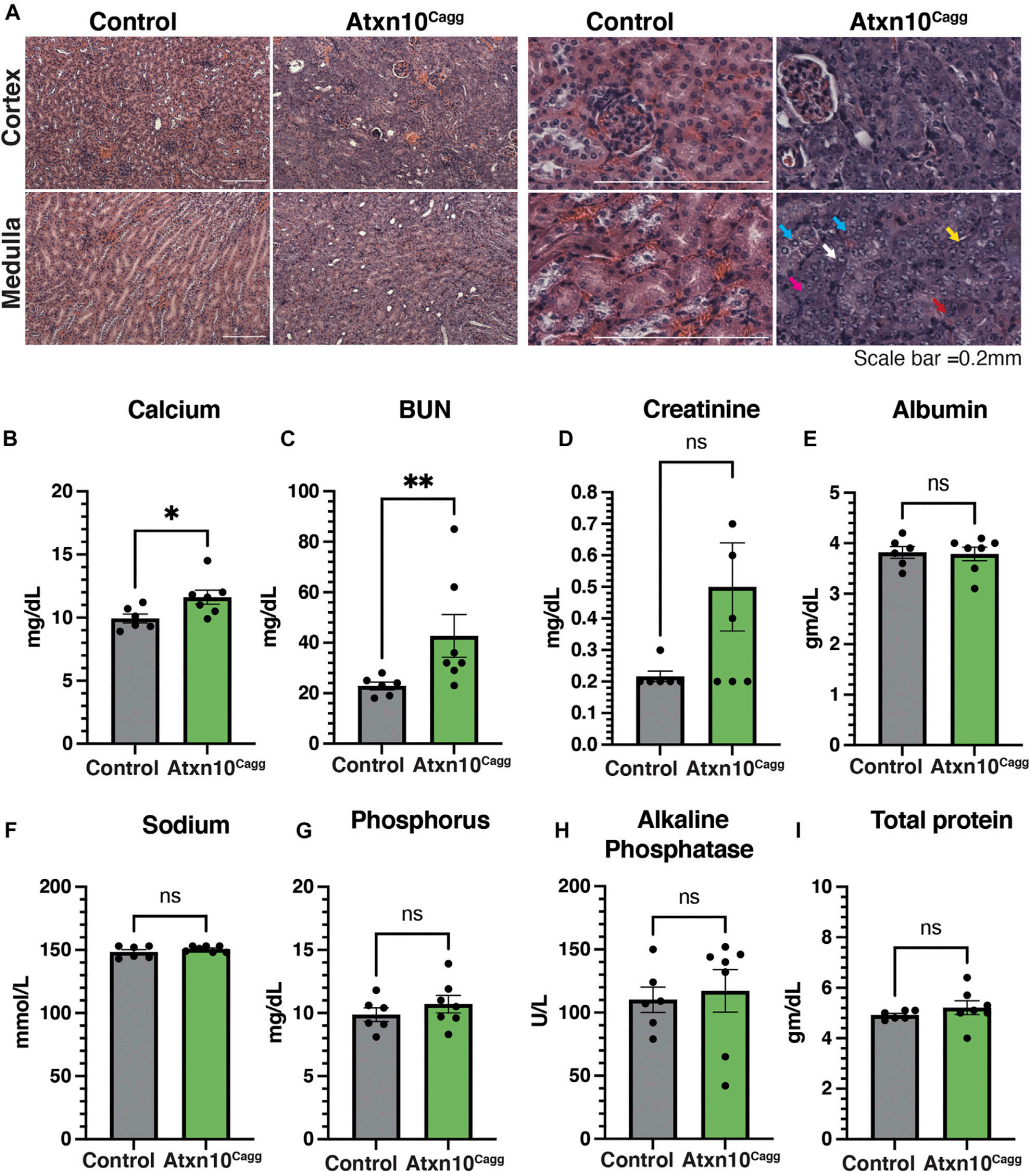
Renal defects associated with ATXN10 loss. **(A)** H&E staining of control **(left)** and *Atxn10* ^*Cagg*^ **(right)** kidneys at 17 days postinduction in the cortex **(top)** and medulla **(bottom)**. Examples of cytoplasmic basophilia, increased nuclear-to-cytoplasmic ratio, open chromatin, distinct nucleoli, and occasional mitotic figures are highlighted by purple, white, blue, yellow, and red arrows, respectively. Blood serum levels of **(B)** calcium, **(C)** BUN, **(D)** creatinine, **(E)** albumin, **(F)** sodium, **(G)** phosphorous, **(H)** alkaline phosphatase, and **(I)** total protein in control (*N* = 6) and *Atxn10* ^*Cagg*^ (*N* = 7) animals at 17 days postinduction. Scale bars = 50 μm. Statistical analysis was performed using unpaired *t*-test for all samples except BUN and alkalkine phosphatase, for which a Mann–Whitney test was performed.

While pathological analyses of the retina, liver, lungs, and spleen (Supplementary Figures S3C–3F) were largely unremarkable, the kidneys presented with histological markers suggestive of a regenerative response to acute tubular injury marked by cytoplasmic basophilia, an increased nuclear to cytoplasmic ratio, open chromatin, distinct nucleoli, and occasional mitotic figures in the proximal tubules (Figure 5A). Despite these histological findings, blood serum analysis revealed that while calcium (Figure 5B) and blood urea nitrogen (BUN) (Figure 5C) levels were significantly increased in *Atxn10* ^*Cagg*^ animals, serum creatinine (Figure 5D, *p* = 0.1), albumin (Figure 5E), sodium (Figure 5F), phosphorus (Figure 5G), alkaline phosphatase (Figure 5H), and total protein (Figure 5I) were not significantly different between the control and *Atxn10* ^*Cagg*^ animals.

### Loss of Ataxin 10 Causes the Pancreatic Epithelium to Become more Progenitor-like and Results in Ectopic Primary Cilia Growth

**FIGURE 6:**
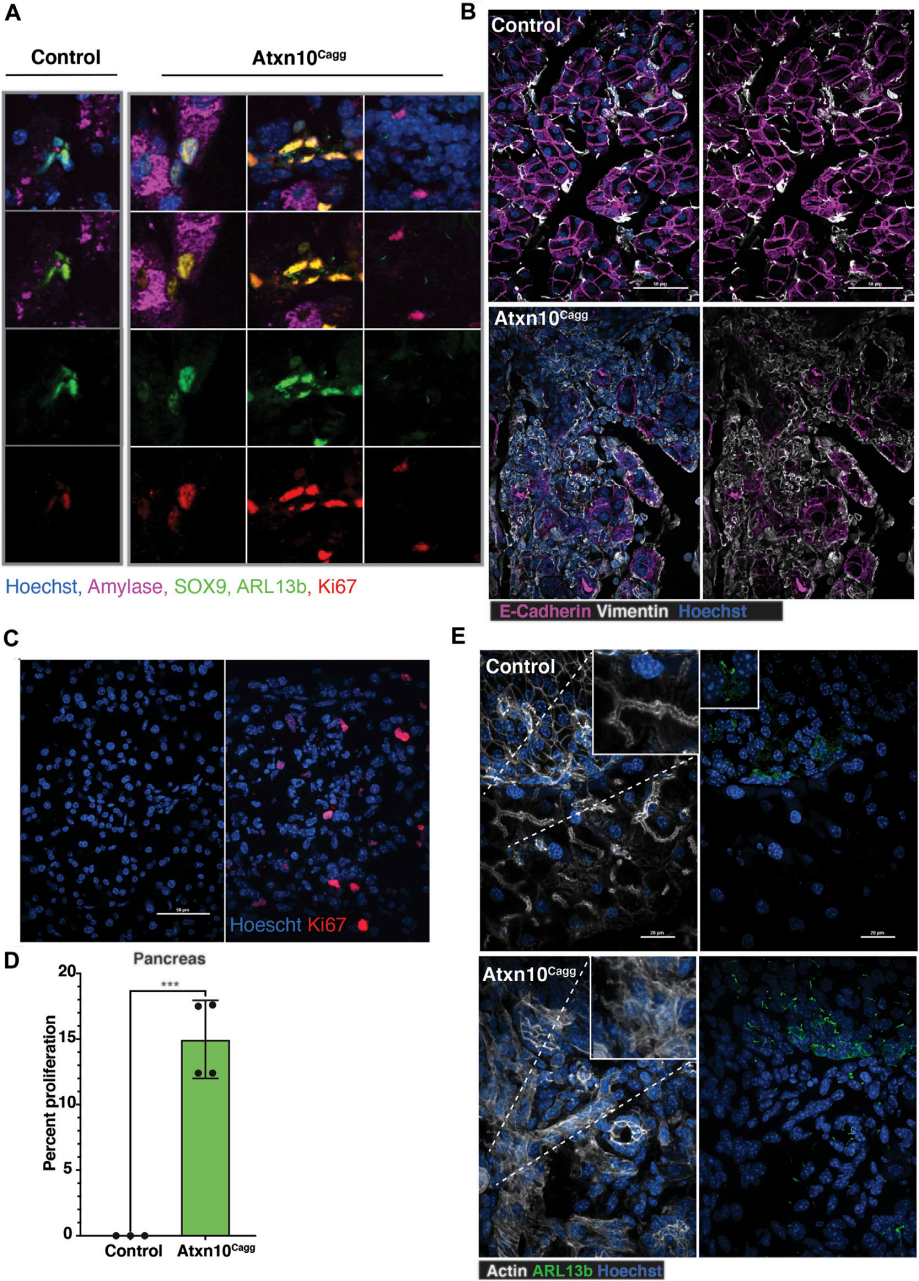
Loss of epithelial characteristics in adult induced *Atxn10* ^*Cagg*^ pancreata. **(A)** Immunofluorescence staining for amylase (purple), Sox9 (green), Arl13b (green), Ki67 (red), and Hoechst (blue) in control and *Atxn10* ^*Cagg*^ pancreata. **(B)** Immunofluorescence staining for E-cadherin (purple), Vimentin (white), and Hoechst (blue) in control **(top)** and *Atxn10* ^*Cagg*^ **(bottom)** pancreas. **(C)** Images and **(D)** quantification of proliferation in the pancreas (*p* = 0.003; *N* = 3 Cre− and 4 Cre+ animals) as shown by Ki67 staining (red) (scale bar = 50 μm). **(E)** Immunofluorescence staining for actin (white), cilia shown by Arl13b staining (green), and Hoechst (blue) in the control **(top)** and *Atxn10* ^*Cagg*^ **(bottom)** pancreata (scale bar = 20 μm).

Within the pancreas, acinar cells can be identified by the presence of amylase while ductal cells express SOX9 (Kopp et al., 2012). In the control pancreata, ductal cells were identified by SOX9-positive nuclei that are frequently also positive for the proliferation marker Ki67. In the *Atxn10* ^*Cagg*^ pancreata, there was an increase in the number of cells that are positive for SOX9 and Ki67 but lacked amylase, suggesting an increase in the number of ductal cells. Intriguingly, there were also cells that are positive for SOX9 and Ki67 that also express amylase that were not present in the controls. In the most severely affected pancreata, a population of ciliated cells that lack SOX9, amylase, and Ki67 are present (Figure 6A). The identity of this cell type is not known, but in a normal pancreas, cilia are restricted to cells in the islets and the ducts. Closer evaluation of *Atxn10* ^*Cagg*^ pancreata showed a substantial increase in mesenchymal cells, as shown by Vimentin-positive staining, accompanied by a decrease in E-cadherin-expressing cells. Furthermore, E-cadherin-positive cells exhibited highly disorganized E-cadherin cellular localization (Figure 6B). Compared with controls, *Atxn10* ^*Cagg*^ pancreata exhibited a significantly higher number of Ki67-positive cells (*p* = 0.0004; *N* = 3 Cre− and 4 Cre+ animals) (Figures 6C,D). Furthermore, *Atxn10* ^*Cagg*^ pancreata exhibit an increase in the density of primary cilia, as marked by the small GTPase ARL13B, in the exocrine region of the pancreas and a disruption to the actin network in the exocrine and endocrine regions (Figure 6E). ARL13B is a small GTPase that localizes to the primary cilium (Caspary et al., 2007). ARL13B-positive cilia were present throughout the islet of the control pancreas, but were not detectable on cells in the exocrine region (Supplementary Figure S4, left). In *Atxn10* ^*Cagg*^ pancreata, the islets exhibited longer cilia, and the exocrine regions of the pancreas had a high density of ciliated cells (Supplementary Figure S4, right). Collectively, these results indicate that in the absence of ATXN10, pancreatic epithelial cells had altered organization and increased proliferation.

### Loss of Ataxin 10 Induces Proliferation and Structural Abnormalities in the Kidney

**FIGURE 7:**
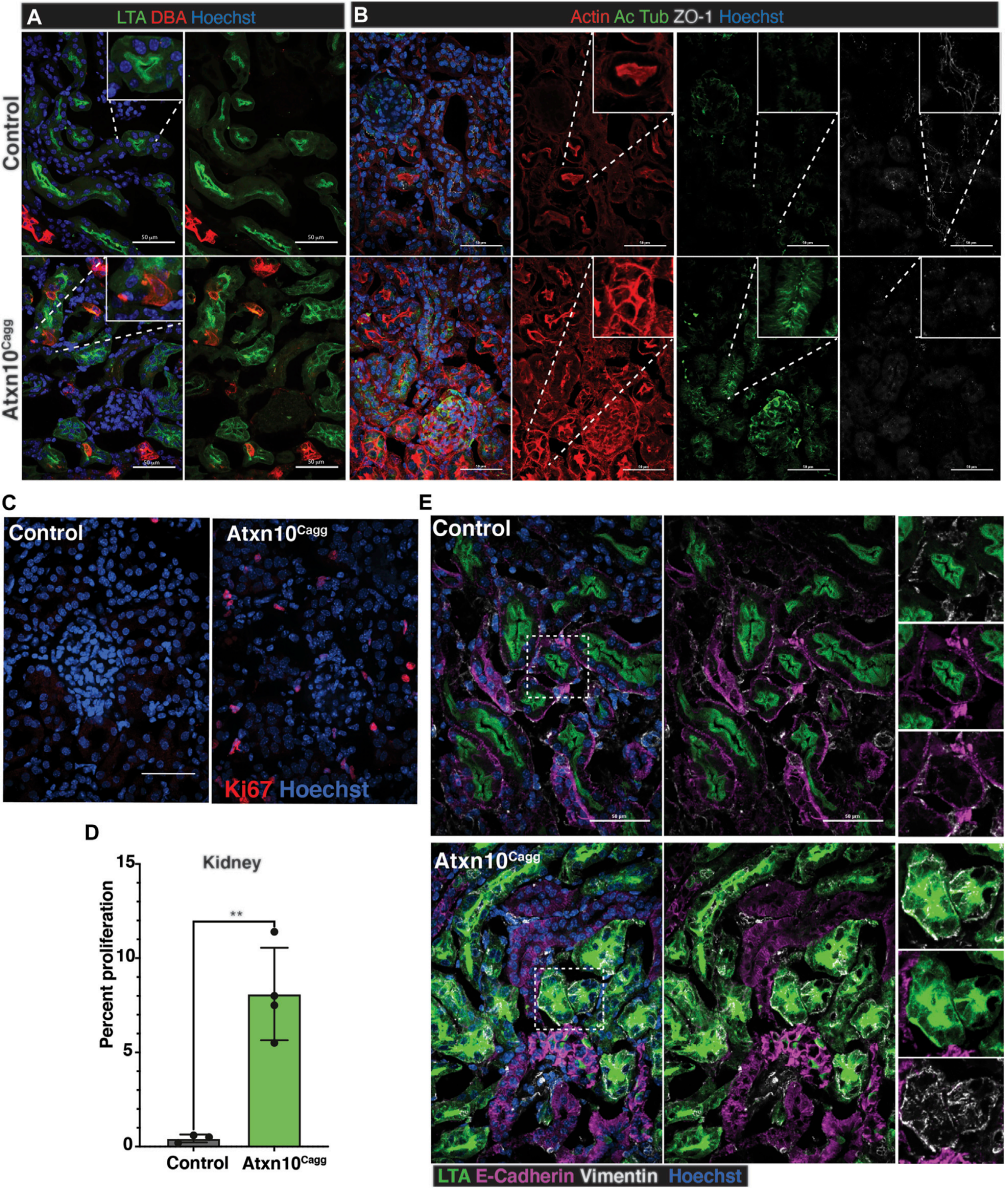
Loss of epithelial characteristics in adult induced *Atxn10* ^*Cagg*^ kidneys. **(A)** Staining for proximal tubule (LTA, green) and collecting duct (DBA, red) in control **(top)** and *Atxn10* ^*Cagg*^ **(bottom)** induced at 8 weeks of age and analyzed at 17 days postinduction. **(B)** Staining for actin (phalloidin, red), cilia (acetylated α-tubulin, green), and ZO-1 (white) in control **(top)** and *Atxn10* ^*Cagg*^ **(bottom)** kidneys. Note punctate cytosolic staining for ZO1 in the inset of the mutant. **(C)** Images and **(D)** quantification of proliferation in the kidneys (*p* = 0.0004; *N* = 3 Cre− and 4 Cre+ animals) as shown by Ki67 (red) staining. **(E)** Immunofluorescence staining for LTA (green), E-cadherin (purple), Vimentin (white), and Hoechst (blue) in control **(top)** and *Atxn10* ^*Cagg*^ **(bottom)** kidneys (scale bar = 50 μm).

Analysis of the kidney by immunofluorescence staining for LTA (proximal tubules) and DBA (collecting tubules/ducts) identified tubule segments in *Atxn10* ^*Cagg*^ animals in which LTA and DBA colocalized, and in many tubules, LTA was no longer restricted to the apical surface of the cells (Figure 7A)*.* Actin staining in *Atxn10* ^*Cagg*^ kidneys further highlighted structural disorganization. Actin organization in control kidneys showed the normal dense actin staining on the brush border on the apical side of the renal epithelium compared with basolateral edges of the cell. In *Atxn10* ^*Cagg*^ kidneys, this staining was no longer enriched at the apical surface, but rather, there was actin accumulation around the entire cellular membrane in many tubules (Figure 7B, red). Furthermore, staining for the presence of cilia with acetylated α-tubulin showed disorganized punctate cilia (Figure 7B, green). To further support the loss of tubule structure in *Atxn10* ^*Cagg*^ kidneys, we examined localization of the tight junction marker ZO-1. Similar to the *in vitro* data, *Atxn10* ^*Cagg*^ samples showed a disorganized appearance of ZO-1 staining in the cytoplasm compared with the expected membrane-associated staining indicative of mature tight junctions (Figure 7B, white). In summary, the *Atxn10* ^*Cagg*^ kidneys exhibited characteristics of disrupted epithelial polarity such as loss of apical localization of LTA, F-actin remodeling, small punctate cilia, and loss of ZO-1 localization at the cell-to-cell contacts. These phenotypes presented between 14 and 17 days following induction shortly before the onset of lethality (Supplementary Figure S5). Furthermore, compared with the control kidneys, *Atxn10* ^*Cagg*^ kidneys exhibited a significantly higher number of Ki67-positive cells (Figures 7C,D, *p* = 0.003; *N* = 3 Cre– and 4 Cre+ animals).

### Loss of Ataxin 10 Results in more Mesenchymal-like Cells in the Renal Tubules

Collectively, the mixed tubule identity paired with loss of apical restriction of LTA, actin remodeling, and loss of ZO-1 at the cell membrane in the *Atxn10* ^*Cagg*^ renal proximal tubules raised the question of whether epithelial cells were becoming more mesenchymal-like. To test this hypothesis, Cre-negative-induced (control) and Cre-positive (*Atxn10* ^*Cagg*^)-induced kidneys were stained for E-cadherin and Vimentin to identify epithelial and mesenchymal cells, respectively. In the control kidneys, E-cadherin staining was observed at the basolateral membrane and at areas of cell-to-cell contact throughout the tubules (Figure 7E). Vimentin staining was adjacent to the E-cadherin-positive basolateral membrane of the renal tubules. In contrast, cells in *Atxn10* ^*Cagg*^ tubules showed LTA-positive staining that is spread diffusely throughout the cell. Furthermore, these LTA-positive cells did not express E-cadherin, but rather were positive for the mesenchymal marker, Vimentin (Figure 7E).

## Discussion

Previous studies of *Atxn10* have focused on its role in SCA10, a pentanucleotide expansion disorder (Matsuura et al., 2000), but there has been limited analysis of the direct consequence of mutations affecting coding regions. Studies highlighting the role of ATXN10 in cell biology have been performed *in vitro* and have centered on its role in cell division. Additionally, studies using the G-LAP-Flp purification strategy in intermedullary collecting duct cells (IMCDs) identified ATXN10 as having indirect interactions with the ciliary transition zone and ciliopathy protein NPHP5 (Torres et al., 2009; Sang et al., 2011). Finally, tandem affinity purification and stable isotope labeling affinity chromatography have also indicated that ATXN10 interacts with the small GTPase ARL13b, which localizes to the primary cilium, further suggesting that ATXN10 may play a role in cilia function or formation (Cevik et al., 2013). Our findings show that ciliogenesis is not overtly affected upon the loss of ATXN10 *in vitro* or *in vivo*. Consistent with previous reports, exogenous expression of ATXN10 in cultured cells showed diffuse localization within the cytoplasm with enrichment at the centrioles and base of the cilium. Previously reported interactions between ATXN10 and NPHP5 further support that ATXN10 is localized at the base of the cilium. We attempted to confirm this localization with the endogenous ATXN10 protein, but none of the available commercial antibodies to ATXN10 were specific for the protein. Thus, the localization has the caveat that it is based on high level exogenous expression. Previous work has shown cell cycle-specific localization of phosphorylated ATXN10 (Tian et al., 2015). Cultured *Atxn10* ^*Cagg*^ renal epithelial cells grown to confluency and then induced for ATXN10 loss adopted a more fibroblast-like appearance and disrupted localization of ZO-1 to the sites of cell-to-cell contact. In addition, we observed defects in chromosomal segregation with a high frequency of micronuclei along with chromosomal bridges that were not present in the controls.

*In vivo*, ATXN10 function is essential for viability. Based on the expression analysis available through MGI and our LacZ reporter analysis, expression of *Atxn10* is broad but is strongly enriched in the developing heart until after E10.5 when expression expands rapidly to cells throughout the embryo. Reflecting the highly localized expression pattern in the early heart, loss of ATXN10 resulted in severe pericardial effusion and ultimately cardiac failure. While *Atxn10* expression is concentrated in the developing heart through E10.5, defects are also observed in the epithelial cells of the neural tube in *Atxn10* ^*KO*^ embryos in which low-level *Atxn10* expression was detected. This is the earliest indication that ATXN10 plays a role in epithelial and endothelial cell maintenance. We did attempt to correlate the expression and localization data generated with the reporter with endogenous expression by *in situ* hybridization, but none of the probes utilized revealed differences between the null mutant and wild-type controls. Thus, the *Atxn10* expression data must be interpreted with this caveat.

To determine if ATXN10 is required in a tissue/cell type-specific manner in the cardiovascular system during embryogenesis, *Atxn10* was deleted in the developing myocardium and endothelium using cTnT-Cre and Tie2-Cre, respectively. Both of these tissue-specific conditional *Atxn10* knockouts result in embryonic lethality. In the case of *Atxn10* ^*Tie2*^ embryos, lethality typically occurred between E11.5 and E13.5. In *Atxn10* ^*Tie2*^ embryos obtained at E10.5, the developing vasculature was present indicating that lethality is not a result of a failure in vasculogenesis. Tie-2 transgene activity is also reported in hematopoetic progenitor cells, and this may also contribute to lethality in these animals (Tang et al., 2010).

Comparatively, *Atxn10* ^*cTnT*^ embryos exhibited cardiac abnormalities similar to those seen in null embryos, but survive longer than *Atxn10* ^*KO*^ or *Atxn10* ^*Tie2*^ embryos. This could be due in part to delayed activation of Cre in this line or to mosaicism in expression of the Cre. Regardless, at E18.5, the hearts of *Atxn10* ^*cTnT*^ embryos displayed reduced ventricular wall thickness reminiscent of the defects observed in *Atxn10* ^*KO*^ embryos at earlier time points.

The importance of ATXN10 is not limited to embryonic development. Using the inducible CaggCre model, loss of ATXN10 in adult animals caused a rapid decline in health and resulted in lethality 3 weeks postinduction. This occurs even with the mosaic deletion observed in most tissues analyzed. Necropsy and histological analysis pointed to moderate to severe pancreatic abnormalities and gastritis paired with reduced food intake and renal abnormalities as the leading cause of lethality. Furthermore, increased cytoplasmic basophilia, was observed in the *Atxn10* ^*Cagg*^ pancreata and kidneys. These phenomena are a result of RNA in the cytoplasm and is indicative of a regenerative epithelial cell or a precursor state in which a cell has recently stopped dividing (Chan, 2014).

The cellular events in multiple tissues associated with this decline were disruption to cell polarity (loss of apical restriction of LTA), loss of epithelial cell-to-cell junctions, downregulation/mislocalization of epithelial markers, such as E-cadherin and zonula occludens (ZO-1), paired with an upregulation of mesenchymal markers, such as Vimentin, and alterations in the actin cytoskeletal architecture (Sheng and Zhuang, 2020). *Atxn10* ^*Cagg*^ kidneys exhibited loss of apical restriction of LTA, dissolution of ZO-1 localization at the membrane, downregulation of E-cadherin paired with an upregulation of Vimentin, and alterations in the actin cytoskeletal network. Collectively, these findings point to EMT in *Atxn10* ^*Cagg*^ kidneys. EMT of renal tubule epithelial cells is associated with the injury and repair process of the kidney (Humphreys et al., 2008). Further indication that the *Atxn10* ^*Cagg*^ kidneys exhibited an injury repair-like process was the increase in Ki67-positive cells. In the kidneys, baseline proliferation is normally very low. Cells begin to acquire Ki67 in the nucleus during the S phase of the cell cycle, and its presence persists throughout the G2 and M phases, and it is degraded in G1 (Miller et al., 2018). Increased proliferation is part of the adaptive repair process of the kidneys, defects in which result in fibrosis (Nadasdy et al., 1994; Sheng and Zhuang, 2020).

In *Atxn10* ^*Cagg*^ pancreata, the exocrine regions also exhibited similar actin misorganization, a loss of E-cadherin, upregulation of Vimentin, and an increase in Ki67-positive cells. In the pancreas, the typical cellular response to an injury is through acinar-to-ductal metaplasia (ADM). Specifically, during ADM, the epithelial pancreatic acinar cells are thought to assume a more progenitor or ductal epithelial cell status (Storz, 2017). Unlike the kidneys, *Atxn10* ^*Cagg*^ pancreata exhibited the added phenomena of an increase in ciliation in the exocrine region. In a normal pancreas, the cell types that are predominantly ciliated reside in the islet and the ducts with the acini not possessing cilia (Augereau et al., 2016). Like *Atxn10* ^*Cagg*^ kidneys, the pancreata also exhibited an increase in Ki67-positive cells. In both of these tissues, baseline proliferation is normally very low.

The rapid decline in health and death of *Atxn10* ^*Cagg*^ animals prevents the observation of longer-term abnormalities. In the congenital model, the disposition toward cardiac abnormalities is likely due to the spatiotemporal expression pattern of *Atxn10.* In the postnatal inducible models, the phenotypes can be broadly classified as a disruption of the epithelial cell function and integrity. In the pancreatic acinar cells, renal epithelial tubules, and glandular epithelium, the epithelial cells exhibit signs of disrupted polarity, loss/mislocalization of epithelial junctional complex proteins, such as E-cadherin and ZO-1, and adoption of a more mesenchymal phenotype as evidenced by the expression of Vimentin. This is strongly suggestive that the epithelium is likely undergoing EMT.

The efficiency of Cagg–CreER-mediated disruption of *Atxn10* expression and recombination was analyzed by PCR in cDNA and in genomic DNA, respectively, from the brain, kidneys, pancreas, and liver. Evaluation of genomic DNA revealed significant, although incomplete, recombination in all tissues. Analysis of recombination in the expressed transcript by rt-PCR mirrored the same level of recombination with the exception of the pancreas and liver. Low recombinase activity in the liver, where there are no overt phenotypes, is not unexpected based on the original characterization of the Cre line (Hayashi and Mcmahon, 2002). The absence of a delta band in the rt-PCR analysis of the pancreas, which displays a severe phenotype, could be attributed to several possibilities including the loss of ATXN10 resulting in cell death. Alternatively, cells originally expressing *Atxn10* are still present but no longer express the gene and were left detecting wild-type *Atxn10* expression only in cells in which recombination did not occur; the mutant transcript is highly unstable in the pancreas and is degraded, or finally, the observed phenotype is a secondary consequence of *Atxn10* loss elsewhere. It is currently unclear as to why specific tissues are preferentially affected. It is probable that if animals lived longer or if tissue-specific Cre mouse lines were used, similar functions of ATXN10 in additional tissues would be uncovered.

Collectively, we showed that ATXN10 is located at the base of the primary cilium, but it is not necessary for ciliogenesis. Furthermore, we showed that ATXN10 is necessary for both embryonic and postembryonic survival with loss in adult animals resulting in an EMT-like progression in the kidneys and pancreas and with cells also undergoing ADM in the pancreas. In none of the adult induced mutants did we observe phenotypes consistent with SCA. This raises the possibility that the petanucleotide repeat in the SCA10 patients may not be due to the loss of ATXN10 protein directly. However, the complication with this assessment is that the mice die rapidly following induction, and this may preclude the presentation of SCA phenotypes.

## Materials and methods

### Generation of Ataxin 10 Mutant Alleles

All animal studies were conducted in compliance with the National Institutes of Health *Guide for the Care and Use of Laboratory Animals* and approved by the Institutional Animal Care and Use Committee at the University of Alabama at Birmingham. Mice were maintained on LabDiet^®^ JL Rat and Mouse/Irr 10F 5LG5 chow. The *Atxn10* ^*KO*^ allele (*tm1a*) was rederived from sperm obtained from the Knockout Mouse Project (KOMP) Repository into C57/B6J strain mice. Mice were maintained on a mixed B6/129 background. *Atxn10* conditional allele (*tm1c*) mice were generated by mating the *Atxn10* ^*KO*^ to FlpO recombinase mice (C57BL/6J), thus, removing the LacZ and Neo cassettes and generating a conditional allele (tm1c; flox). Progeny that contained the recombined allele were crossed off of the FlpO line and bred to respective Cre recombinase males. Here we refer to these alleles as the *tm1a* (*Atxn10* ^*KO*^), *tm1c* (*Atxn10* ^*flox*^), and *tm1d* (*Atxn10* ^*Cagg*^, *Atxn10* ^*cTnT*^, or *Atxn10* ^*Tie2*^) alleles. Primers used for genotyping are as follows: 5′-GAC​TTT​TGG​CAC​CAC​ACA​GC-3′, 5′-GTG​GAA​GGG​CTG​AAA​ACT​GG-3′, 5′-TCG​TGG​TAT​CGT​TAT​GCG​CC-3′, 5′-ATC​ACG​ACG​CGC​TGT​ATC-3′, and 5′-ACA​TCG​GGC​AAA​TAA​TAT​CG-3′. Primers used for rt-PCR analysis of the recombined floxed allele (delta): forward primer in Exon 2 5′-CTG​GCT​TCC​AGT​CTG​CAG​CTA​ATC-3′ and the reverse primer in Exon 6 5′-GTT​GCT​GAG​TTT​GCC​ATA​CAT​AGC​TTC​C-3′.

### Generation and Transfection of Ataxin 10 Expression Constructs

The *MmAtxn10* coding sequence was cloned into the pEGFP-N1 vector (Clontech) using primers designed with XhoI and AgeI restriction sites. *MmAtxn10*-EGFP-N1 plasmids were transfected into cells using *Trans*IT^®^-2020 DNA per manufacturer guidelines (Mirus, MIR5404).

### Generation of cDNA From Tissues

RNA was isolated from wild-type and mutant brain, liver, pancreas, and kidneys via Trizol extraction. cDNA was generated from 50 mg of cDNA using SuperScript IV reverse transcriptase as per the instructions of the manufacturer.

### Embryo Isolation

Timed pregnancies were established with embryonic timepoint of E0.5 being noted at noon on the morning of observing the copulatory plug. To isolate embryos, pregnant females were anesthetized using isoflurane followed by cervical dislocation. Embryonic tissues or whole embryos were isolated and fixed in 4% paraformaldehyde (Sigma PFA, 158127) in PBS.

### β-Galactosidase Staining

For whole mount or slice β-galactosidase staining, samples were fixed (0.2% glutaraldehyde (Sigma), 5 mM of EGTA, and 2 mM of MgCl_2_ in 1X PBS) at 4°C for 40 min. Samples were rinsed three times for 15 min at 4°C (0.02% Igepal, 0.01% sodium deoxycholate, and 2 mM MgCl_2_ in 1X PBS). Samples were immersed in staining solution overnight in the dark at 37°C (1 mg/ml X-gal, 0.02% Igepal, 0.01% sodium deoxycholate, 5 mM potassium ferricyanide, 5 mM potassium ferrocyanide, and 2 mM MgCl_2_ in 1X PBS). Samples were postfixed in 4% PFA and stored at 4°C. Embryos were imaged using a Nikon SMZ800 stereo microscope. Sections were counterstained using Nuclear Fast Red (Sigma).

### Isolation of Mouse Embryonic Fibroblasts

Embryos were isolated at either E9.5 (*Atxn10* ^*KO/KO*^) or E13.5 (conditional lines). Following the removal of the liver (E13.5 only) and head, embryos were mechanically dissociated and cultured in DMEM (Gibco, 21063-021) supplemented with 10% fetal bovine serum, 1X penicillin and streptomycin, 0.05% primocin, and 3.6 µl/0.5 L β-mercaptoethanol. Cilia formation was induced using media containing 0.5% FBS.

### Primary Kidney Epithelium Cell Culture

Mice were anesthetized with isofluorane followed by cervical dislocation. Kidneys were removed and mechanically dissociated. Resulting minced tissue was filtered through a 70-μm cell strainer. Tubules were cultured in DMEM (Gibco, 11039-021) supplemented with 5% FBS, epidermal growth factor (recombinant human, 10 ng/ml), insulin (recombinant human, 5 μg/ml), hydrocortisone (36 ng/ml), epinephrine (0.5 μg/ml), Triiodo-L-thyronine (4 pg/ml), and transferrin (recombinant human, 5 μg/ml) (Growth Medium 2 Supplement Pack, PromoCell, C-39605).

### Pathology and Histology

Mice were anesthetized with 0.1 ml/10 g of body weight dose of 2.0% tribromoethanol (Sigma Aldrich, St. Louis, MO, USA) and transcardially perfused with PBS followed by 4% paraformaldehyde. Tissues were postfixed in 4% PFA overnight at 4°C, cryoprotected by submersion in 30% sucrose in PBS for 16–24 h, then embedded in OCT, and cryosectioned for immunofluorescence, and hematoxylin (Fisher Chemical, SH26-500D) and eosin (Sigma-Aldrich, HT110132-1L) staining was performed. Pathological and histological analyses for *Atxn10* ^*Cagg*^ pancreata, kidneys, spleen, retina, lungs, and liver were performed by the Comparative Pathology Lab (UAB) as follows. Briefly, mice were necropsied, and tissues were fixed in 10% neutral-buffered formalin overnight. Tissues were prosected and processed, then 5 µM sections were stained with hematoxylin and eosin. Slides were evaluated for tissue histopathology by a board-certified veterinary pathologist in blinded fashion.

### Immunofluorescence Microscopy

Ten (10)-micrometer tissue sections were used for immunofluorescence microscopy. For staining MEFs, cells were grown on 0.1% gelatin-coated glass coverslips until confluent, then serum starved using DMEM containing 0.5% FBS for 24 h to induce cilia formation (Breslow and Nachury, 2015). Sections were fixed with 4% PFA for 10 min, permeabilized with 0.1% Triton X-100 in PBS for 8 min and then blocked in a PBS solution containing 1% BSA, 0.3% TritonX-100, 2% (vol/vol) normal donkey serum, and 0.02% sodium azide for 1 h at room temperature. Primary antibody incubation was performed in blocking solution overnight at 4°C. Primary antibodies include acetylated α-tubulin (Sigma, T7451) direct conjugated to Alexa 647 (Invitrogen, A20186) and used at 1:1,000, Arl13b (Proteintech, 1771-1AP, 1:500), PECAM1 (Abcam, ab7388, 1:250), E-cadherin (Abcam,ab11512, 1:300), Phalloidin (Invitrogen, A12380 or A12379, 1:300), Ki67 conjugated to PE, (Thermofisher, 12-5698-80, 1:300), cTnT (DSHB, RV-C2, 1:300), ZO-1 (R40.76, 1:2), Vimentin (Abcam, ab92547, 1:300), Amylase (Abcam, ab189341, 1:1,000), and Sox9 (Abcam, ab185230, 1:300), Fluorescein-labeled *Lotus tetragobolobus* lectin/LTA (Vector Laboratories, FL-1321, 1:250), and rhodamine-labeled *Dolichos biflorus* agglutinin (DBA) (Vector Laboratories, RL-1032, 1:250). Cryosections were washed with PBS three times for 5 min at room temperature. Secondary antibodies diluted in blocking solution were added for 1 h at room temperature. Secondary antibodies included donkey-conjugated Alexa Fluor 647, 488, and 594 (Invitrogen, 1:1,000). Samples were washed in PBS and stained with Hoechst nuclear stain 33258 (Sigma-Aldrich) for 5 min at room temperature. Coverslips were mounted using SlowFade Diamond Antifade Mountant (Life Technologies). All other fluorescence images were captured on Nikon spinning-disk confocal microscope with Yokogawa X1 disk, using Hamamatsu flash4 sCMOS camera. The 60× apo-TIRF (NA = 1.49), 40× plan fluor (NA = 1.3), or 20× plan fluor multi-immersion (NA = 0.8) objectives were used. Images were processed using Nikon’s Elements or Fiji software.

### Tamoxifen Cre Induction

Recombination of the conditional allele was induced in *Atxn10* ^*flox/flox*^; *CAGG-cre* ^*ERT2*^ mice at 6 and 8 weeks old by a single intraperitoneal (IP) injection of 9 mg of tamoxifen (Millipore Sigma, T5648) per 40 g (body weight) in corn oil. Induction of cell lines was achieved by exposure to media supplemented with 1 mM 4-hydroxytamoxifen for 24 h.

### Statistics

Calculations were performed using Graphpad Prism and Microsoft Excel. Specific tests used are indicated in the figure legends with significance indicated as follows: *p ≤ 0.05, **p ≤ 0.01, and ***p ≤ 0.001. Error bars indicate standard deviation.

## Data Availability

The raw data supporting the conclusions of this article will be made available by the authors, without undue reservation.
