# Supplementary figures and images for: ATXN10 Is Required for Embryonic Heart Development and Maintenance of Epithelial Cell Phenotypes in the Adult Kidney and Pancreas
Source: Front Cell Dev Biol. 2021 Dec 14;9:705182. doi: 10.3389/fcell.2021.705182 (PMC8712648; doi:10.3389/fcell.2021.705182)

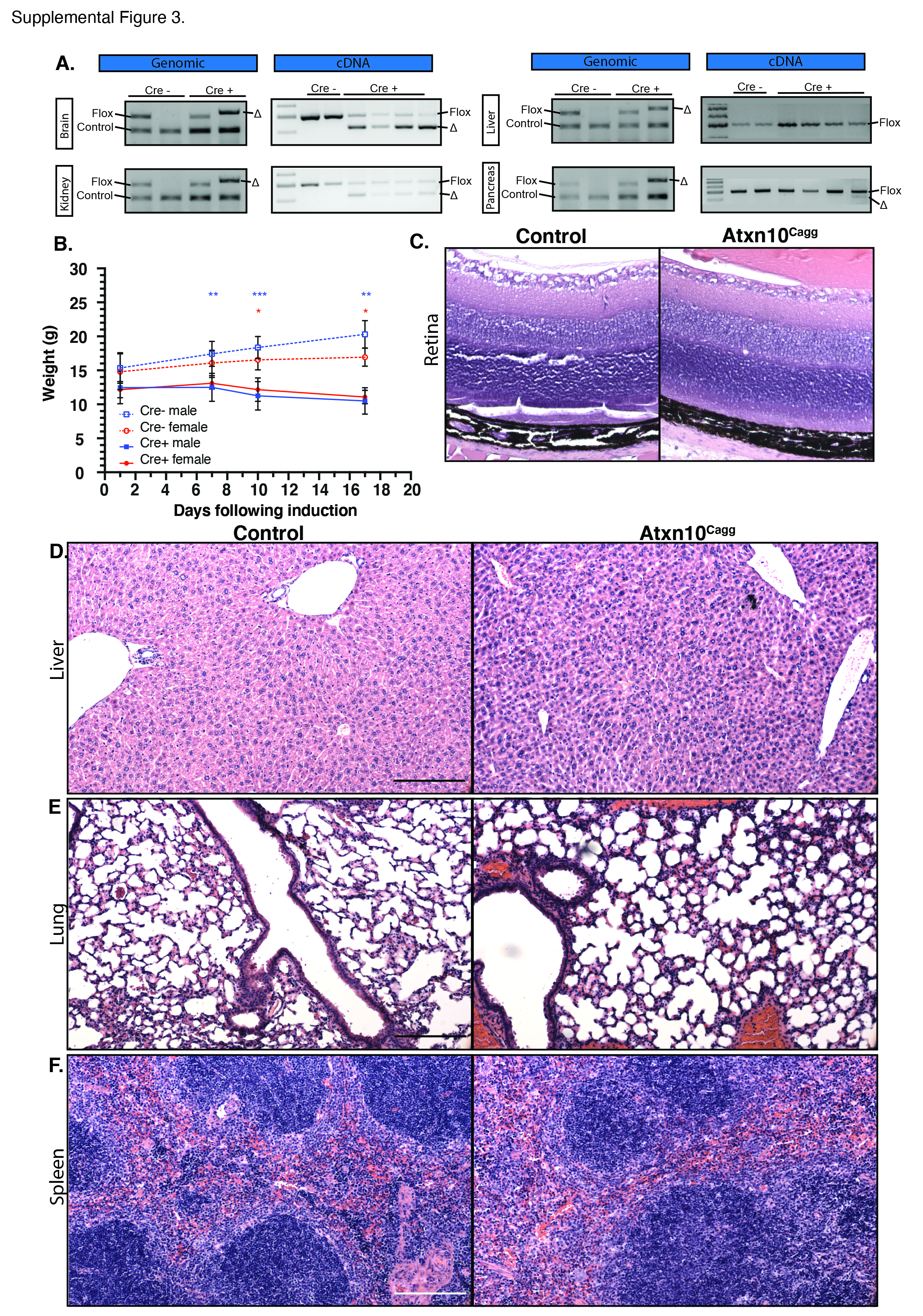

Supplement: Supplementary file 1 [file Image3.TIF]

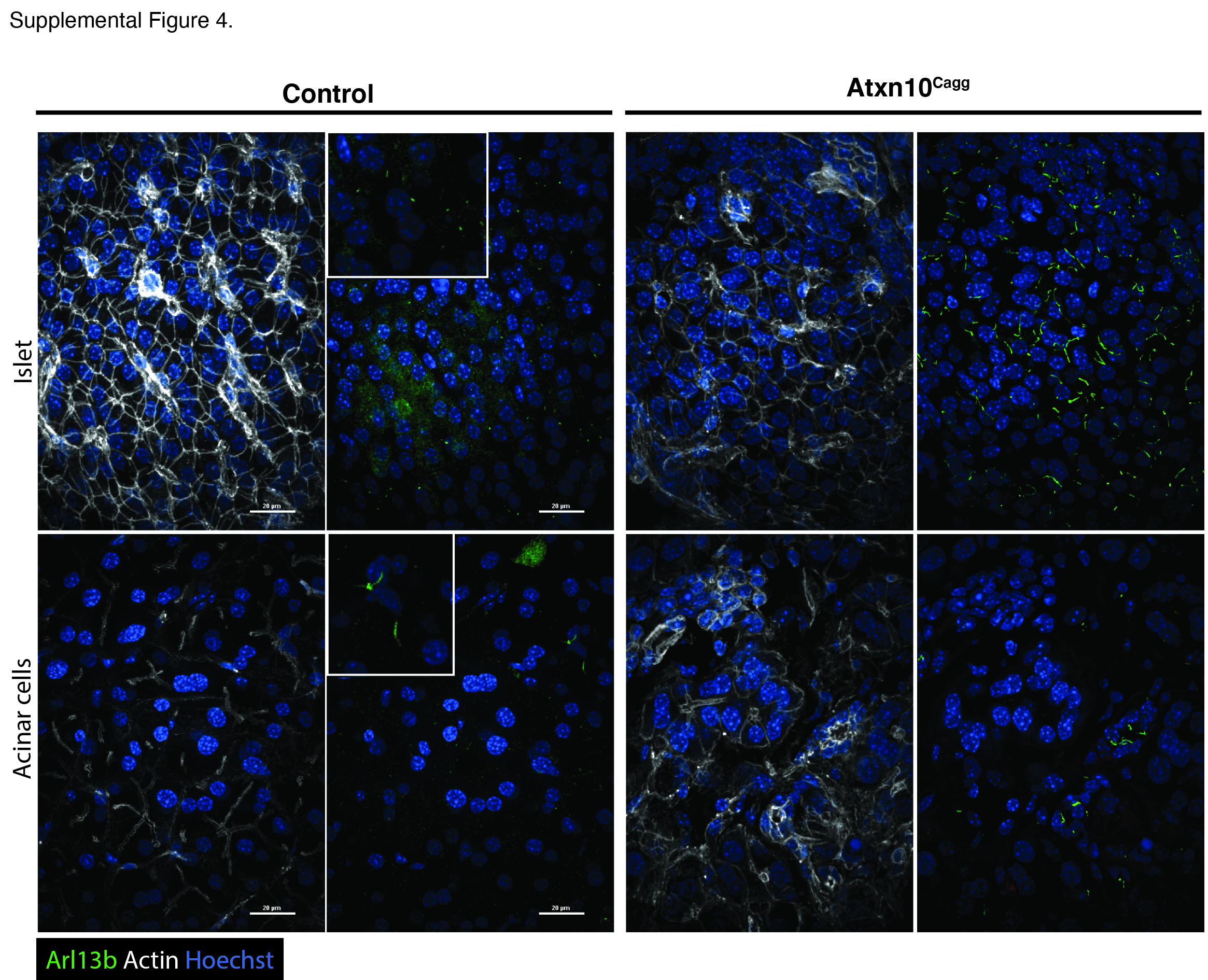

Supplement: Supplementary file 2 [file Image4.TIF]

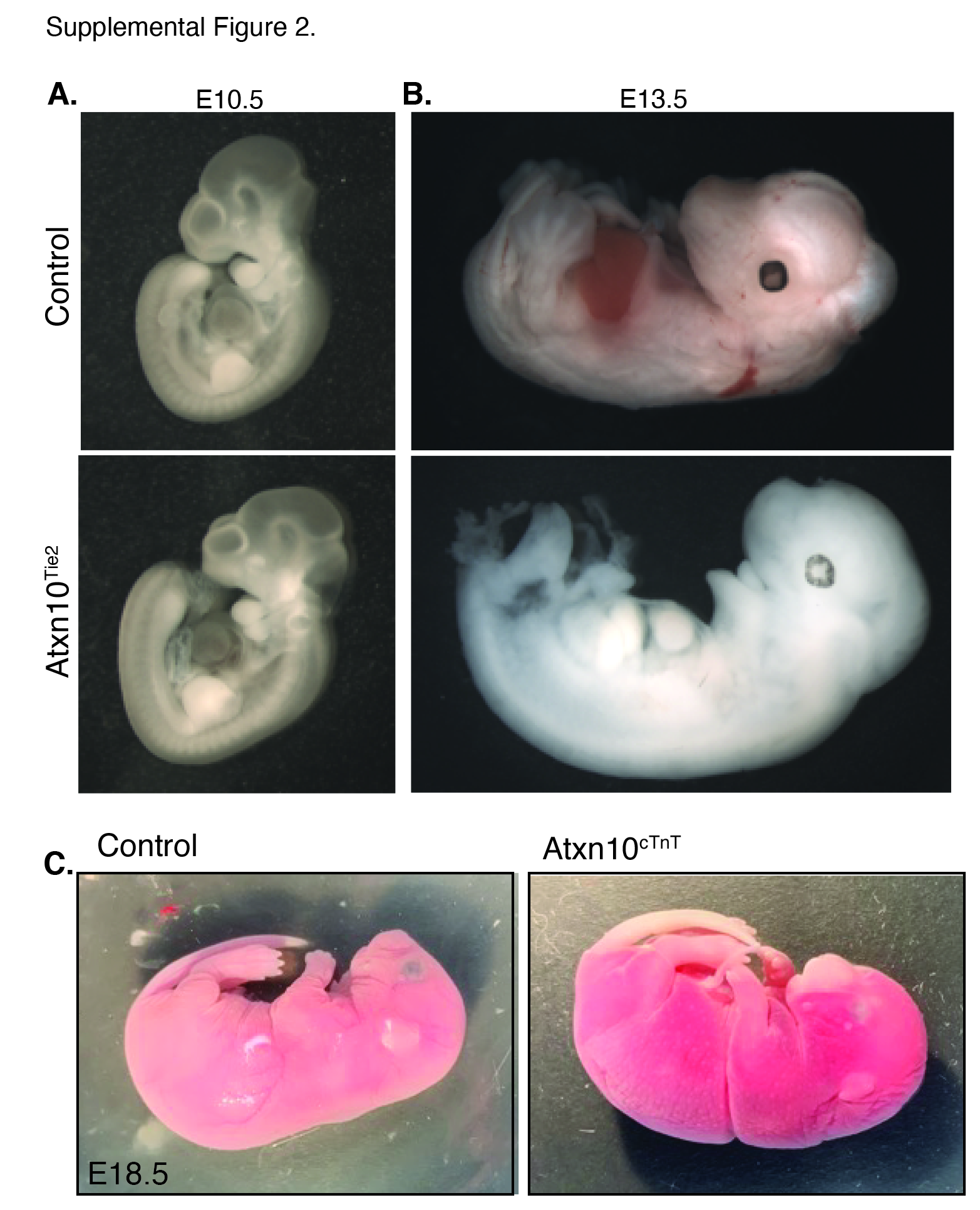

Supplement: Supplementary file 3 [file Image2.TIF]

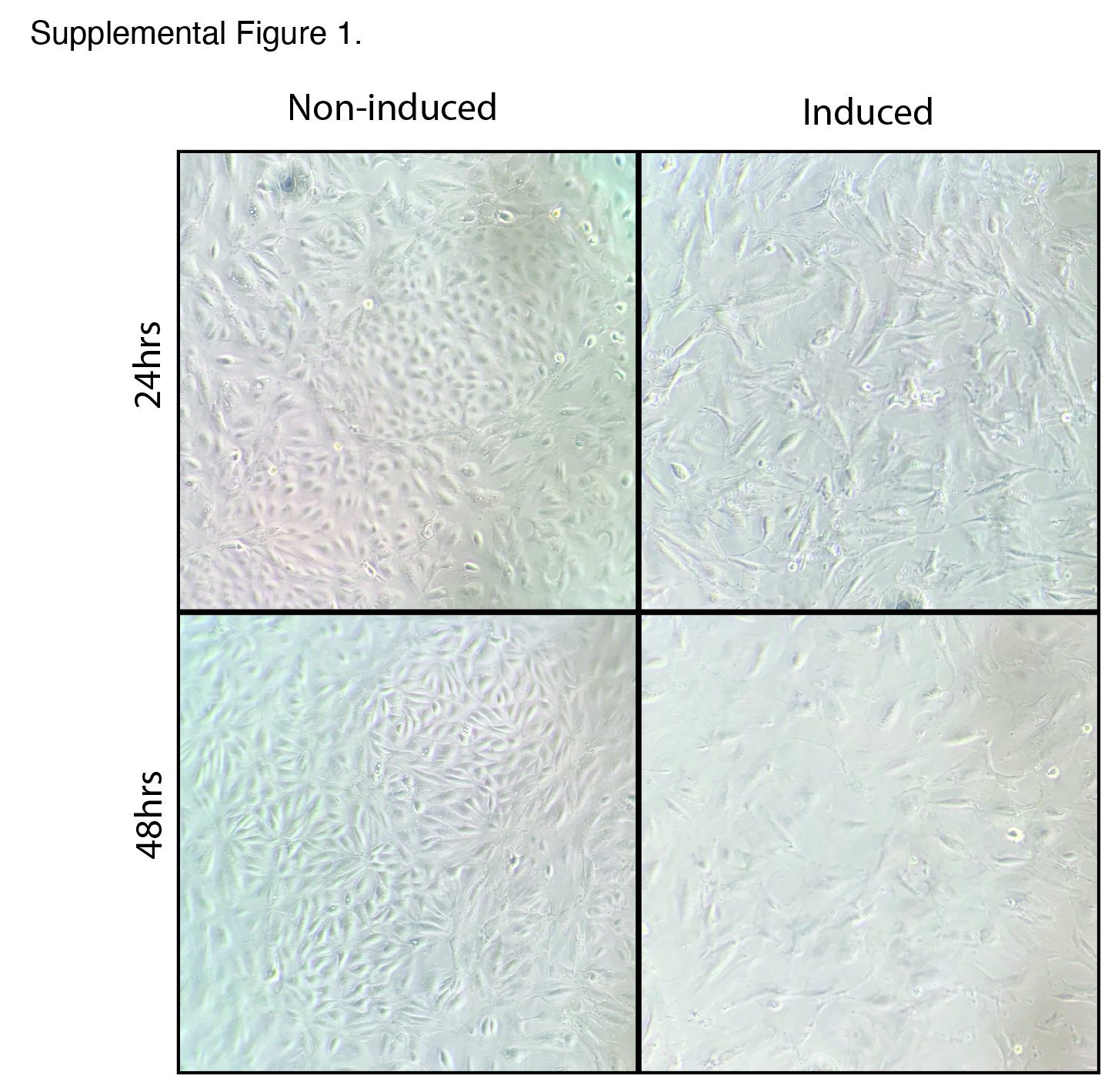

Supplement: Supplementary file 4 [file Image1.TIF]

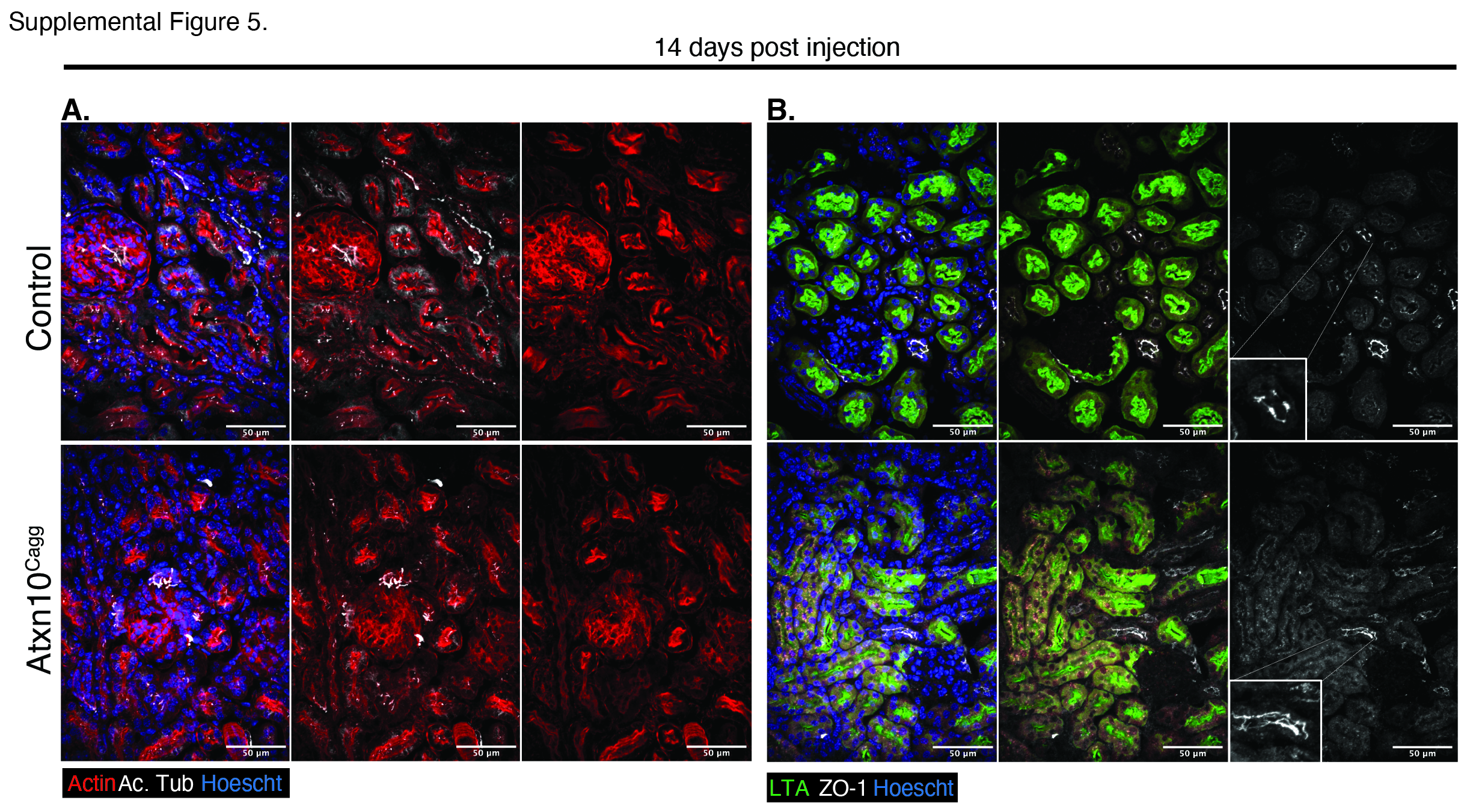

Supplement: Supplementary file 5 [file Image5.TIF]
